# Structural characterization and biological activities of polysaccharide iron complex synthesized by plant polysaccharides: A review

**DOI:** 10.3389/fnut.2022.1013067

**Published:** 2022-09-30

**Authors:** Yongshuai Jing, Shilin Zhang, Mingsong Li, Ruijuan Zhang, Hao Zhang, Yuguang Zheng, Danshen Zhang, Lanfang Wu

**Affiliations:** ^1^College of Chemistry and Pharmaceutical Engineering, Hebei University of Science and Technology, Shijiazhuang, China; ^2^College of Pharmacology, Hebei University of Chinese Medicine, Shijiazhuang, China

**Keywords:** polysaccharide iron complex, preparation methods, structural characterization, biological activities, clinical application

## Abstract

Iron deficiency anemia can lead to a variety of functional disorders, which is one of the highest incidence of nutrient deficiency diseases. The direct addition of iron to food will not only brings difficulties to the production of products, but also brings damages to human body. In recent years, international studies have shown that polysaccharide iron complex (PIC) not only has a variety of pharmacological activities of polysaccharide itself, but also has the function of supplementing iron, so it is a good iron supplement. With the advantages of good solubility, high iron content, low gastrointestinal irritation and high bioavailability, PIC is an effective iron supplement for iron deficiency anemia and has attracted more and more attention. In this paper, the different preparation methods, structural characterization, biological activities and clinical applications of PIC synthesized by natural polysaccharides from plant were reviewed, in order to provide theoretical basis for the development and application of PIC.

## Introduction

Iron is an essential micronutrient and an important component of metalloproteins (hemoglobin, myoglobin, ferritin, transferrin, cytochrome, etc.). It is a basic auxiliary group for regulating, activating and controlling many enzyme reactions and plays a key role in cellular transformation such as DNA synthesis, oxygen transport and energy metabolism (1). Insufficient iron intake from diet or poor gastrointestinal iron uptake from food cause iron deficiency, the most prevalent nutritional problem in the world, which can give rise to some dysfunctions such as anemia, impaired immune system, or pregnancy complications. The solution is enhancement of iron intake, either by supplementation or fortification (2–5).

The first generation of inorganic iron supplements is represented by ferrous sulfate. Although iron content is high and absorption is good, there are adverse reactions such as abdominal discomfort, nausea, loss of appetite, diarrhea and headache. The second generation of iron supplements are soluble small molecular organic iron salts, such as ferrous lactate. Compared with the first generation of iron supplements, the absorption rate and bioavailability of iron ions in improve. But the property of ferrous salt is unstable, production and storage is more difficult, easy to produce peculiar smell. Different from the previous two generations of oral iron supplements, the third generation of iron supplements is intravenous injection, mainly organic iron complexes, such as polysaccharide iron complex (PIC), heme iron, polypeptide iron chelate, iron-rich yeast, etc. The toxicity and side effects of the third generation iron supplements are smaller, among which, polysaccharide iron complex has become a hot research spot at present because of its suitable complexation stability, good tolerance, high bioavailability, high iron content and low production cost (6). At present, the iron supplements of polysaccharide iron complex in the world can be divided into dextran iron, sucrose iron, sodium iron gluconate, carboxyl maltose iron, polysaccharide superparamagnetic iron oxide nanoparticles, isomaltose iron, chitosan iron and plant polysaccharide iron (7). And many kinds of iron supplements have been put into production and use. Oral polysaccharide iron supplements include lifeineng polysaccharide iron complex capsule and hongyuanda polysaccharide iron complex capsule. Polysaccharide iron complexes that can be administered parenterally include dextran iron, sodium iron gluconate, sucrose iron, carboxyl maltose iron, polysaccharide superparamagnetic iron oxide nanoparticles, isomaltose iron 1000, etc (8). The iron complex of chitosan and plant polysaccharide is still in the research stage, and there are few products on the market (9).

**Figure 1:**
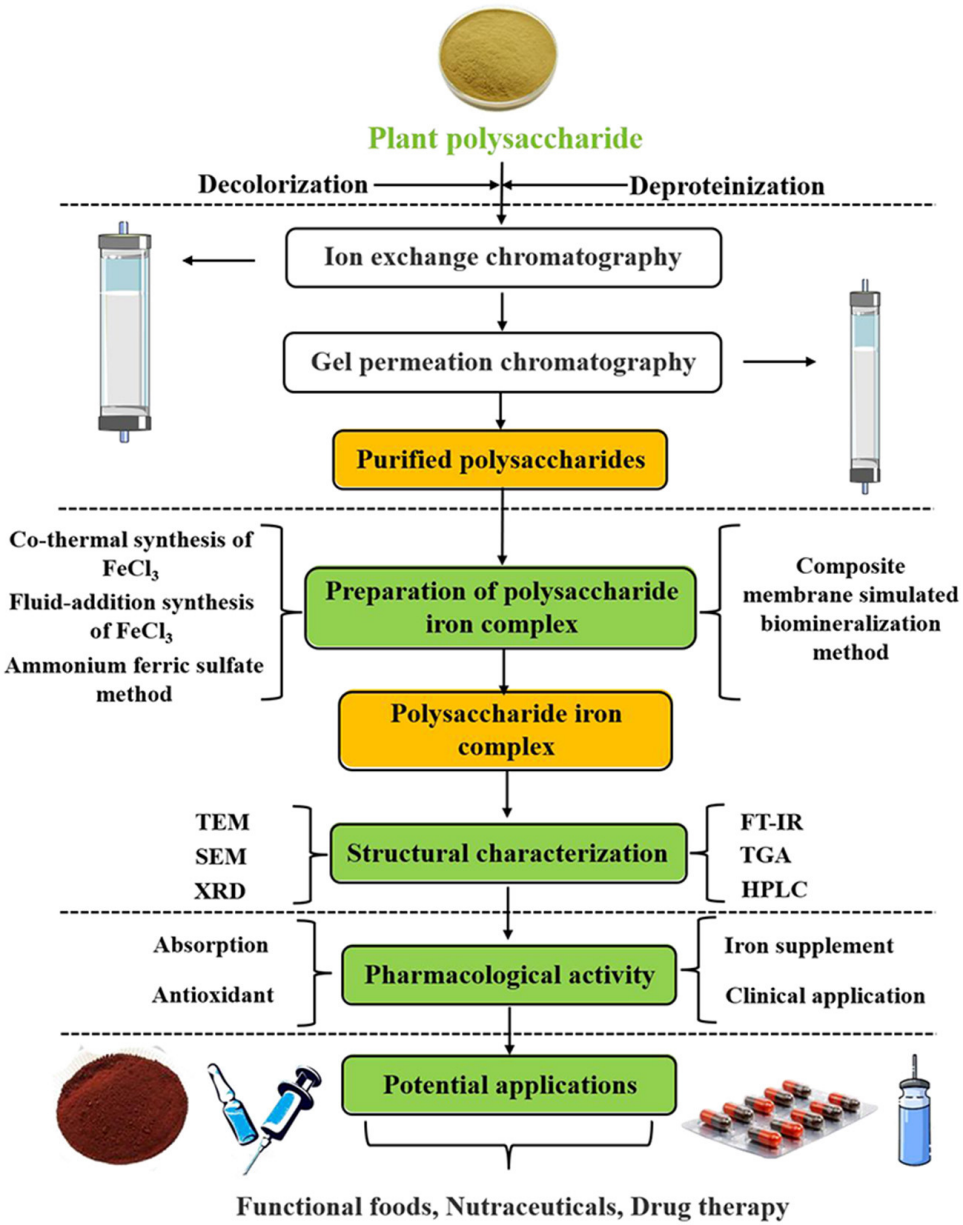
Overall research idea chart of PIC.

Polysaccharide is a carbohydrate composed of more than 10 monosaccharides. It has many biological activities, such as regulating blood sugar, lowering blood pressure, promoting blood circulation, immune regulation. It can also be absorbed and used by the body, so as to play the corresponding effect (10). It is found that PIC has a significant effect on improving iron deficiency anemia. The PIC with polysaccharide as carrier not only retained the activity of polysaccharide and metal ions, but also enhanced their activity (11). As a new kind of iron supplement, PIC has the advantages of small side effects, stable coordination, good solubility, especially in high concentration, non-toxic and so on (12). The synthesis of a new organic iron complex from plant polysaccharides opens up an innovative way for iron supplementation and iron polysaccharide application. In addition, polysaccharide iron complex shows more beneficial pharmacological activities, such as antioxidant and antitumor properties (13). At present, the synthesis methods of PIC include chemical synthesis and composite membrane simulated biomineralization. Chemical synthesis method is still the mainstream, which the advantage are availability of raw materials, short production cycle and easy quality control (14). In this paper, the preparation technology, structural characteristics and pharmacological activity of PIC were reviewed in order to provide a theoretical basis for the development and application of PIC. The overall research idea of PIC is shown in Figure 1.

## Preparation of PIC

PIC is a compound formed by complexing ferric ions with polysaccharide as carrier and is composed of glycosyl and aglycon (Fe^3+^) (9). The glycosyl can be dextran, dextrin, plant polysaccharide, etc. The aglycon is Fe^3+^ and does not exist in a free state, so they will not cause intestinal discomfort in humans. It is absorbed and utilized after being reduced to divalent iron in organisms (14). The iron chelated by polysaccharides is mainly Fe^3+^, which is discussed in this paper. At present, the preparation methods of PIC mainly include: co-thermal synthesis of FeCl_3_ method, fluid-addition synthesis of FeCl_3_ method, ammonium ferric sulfate method and composite membrane simulated biomineralization method. The co-thermal synthesis of FeCl_3_ is the most widely used in the preparation of iron complex of plant polysaccharides (7). However, ammonium ferric sulfate method and composite membrane simulated biomineralization method are hardly applied to the preparation of plant polysaccharide iron complex, and there are only a few reports on the preparation of chitosan iron complex. Therefore, their preparation technology was also reviewed in this paper, which will provided a new idea and method for the preparation of plant polysaccharide iron complexes.

### Chemical synthesis

#### Co-thermal synthesis of FeCl_3_ method

**Figure 2:**
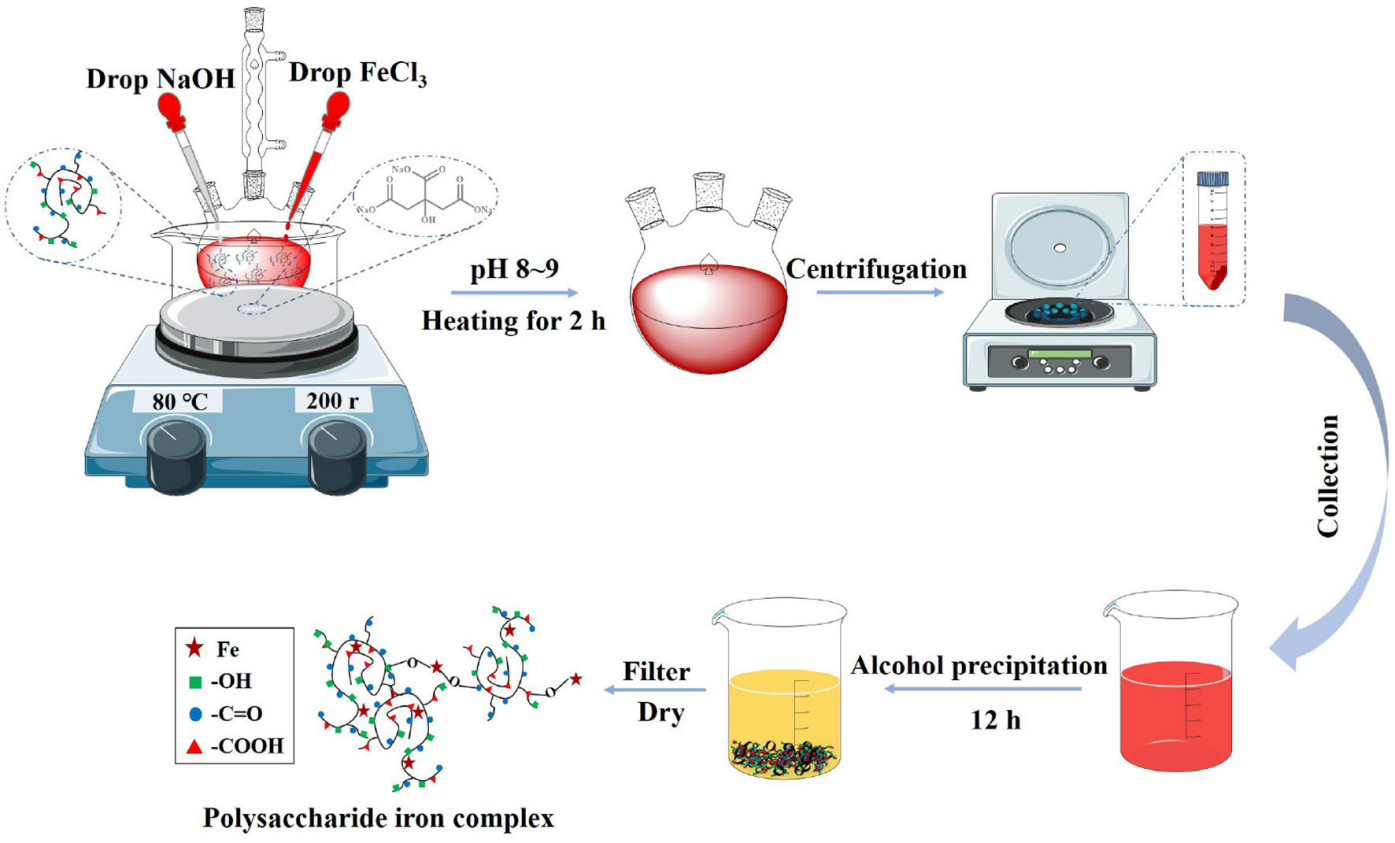
Preparation of PIC by ferric chloride co thermal synthesis.

PIC was prepared by co-thermal synthesis of FeCl_3_ method according to the previous literature (15–23). A certain amount of polysaccharide is dissolved in distilled water to prepare a polysaccharide solution with a certain concentration. Sodium citrate was added to the polysaccharide solution and mixed evenly, and the mixed solution was heated and kept at 65–70°C. Then the mixed solution was added to ferric chloride solution (2 mol/L) dropwise andstirred continuously. The pH was adjusted to 8.0–9.0 by sodium hydroxide solution (2 mol/L). Then ferric chloride solution and sodium hydroxide solution were added to that solution, and when a reddish brown insoluble precipitate appeared in the reaction solution, the dropwise addition was stopped. The reaction solution was kept at 65–80°C for at least 1 h. Then the solution was centrifuged at 8,000 rad/s for 10 min. The supernate was precipitated with absolute ethanol. There were also methods to show that a certain amount of sodium carbonate was added after adding sodium citrate, and the above steps were repeated below. The specific synthesis process was shown in Figure 2. Researchers used this method to prepare a variety of PIC, and the main reaction conditions are shown in Table 1.

**Table 1:** Main reaction conditions for preparation of PIC by ferric chloride co thermal synthesis.

**No**.	**Source of polysaccharides**	**Main reaction conditions**	**Iron content (%)**	**Ref**.
1	*Codonopsis pilosula*	The optimum synthesis conditions for CPPI were pH 8.9, temperature 70.30°C and the ratio of citric acid to CPPI of 2.95.	28.40	(13)
2	*Hypsizygus marmoreus*	Chelation time was 5.5 h, the mass ratio of HMP to iron (III) was 4.5:1 (mg/mg), initial mass concentration of iron (III) was 5.2 mg/mL and pH was 2.1.	86.69	(15)
3	*Taraxacum*	The reaction temperature 60°C, pH 8.51, reaction time 1 h and sodium citrate to Taraxacum polysaccharides was 0.50.	24.0–41.0	(16)
4	*Agaricus blazei*	The reaction temperature 70°C, pH 8.00, reaction time 2 h and sodium citrate to *Agaricus blazei* polysaccharides was 0.50.	15.7	(17)
5	*Polygonum multiflorum*	The reaction temperature 70°C, pH 7–8, reaction time 1 h and sodium citrate to P*olygonum multiflorum* polysaccharids was 0.50.	15.08	(18)
6	*Rehmannia glutinosa* Libosch	The reaction temperature 80°C, pH 8.0, reaction time 1.05 h and sodium citrate to *Rehmannia glutinosa* Libosch polysaccharides was 1.45.	16.03	(19)
7	*Inonotus obliquus*	The ratio of IOPS and FeCl_3_•6H_2_O was 3:5 (w/w), the pH value of alkali solution was 10, the reaction temperature was 30°C and the reaction time was 6 h.	19.40	(20)
8	*Astragalus membranaceus*	The reaction temperature 89.46°C, pH 8.16, reaction time 46.04 min and ratio of catalyst to APS 0.75.	21.08–21.24	(21)
9	*Ulva pertusa*	The reaction temperature 70°C, pH 8.51, reaction time 2 h and sodium citrate to *ulva pertusa* polysaccharides was 0.25.	22.63–28.67	(22)
10	Soybean	The reaction temperature 68.9°C, pH 8.89, reaction time 1.47 h and sodium citrate to SSPS was 0.52.	14.53–15.61	(23)
11	*Enteromorpha prolifera*	The reaction temperature 60°C, pH 8.56, reaction time 1 h and sodium citrate to PEs was 0.15.	20.71–20.99	(24)

Co-thermal synthesis of FeCl_3_ is the earliest preparation method used to synthesize dextran iron complex, and now it is mostly used to prepare plant polysaccharide iron complex (8). The FeCl_3_, sodium citrate and NaOH used in this method are all easily available and cheap reagents, so the synthesis cost of this method is low. At the same time, many studies showed that the reaction time of this method was about 1–2 h, the preparation time was short, the preparation efficiency was high and the reaction conditions were easy to control (24). However, due to the use of chemical reagents, this method also has the disadvantages of environmental pollution and poor recycling.

#### Fluid-addition synthesis of FeCl_3_ method

The principle of this method is that under alkaline conditions, ferric iron (Fe^3+^) is polymerized through oxygen bridge or hydroxyl bridge to form ferric citrate polymer, and a large amount of citric acid is free. Finally, only about 17% citric acid is complexed on the surface of polymeric iron core. Chen et al. (25) synthesized angelica PIC by iron-trichloride flow addition synthesis method, and washed it successively with ethanol, methanol and ether of different concentrations, and finally obtained the red-brown powdered *angelica* PIC through vacuum drying. Fluid-addition synthesis of FeCl_3_ is similar to the co-thermal synthesis of FeCl_3_ in raw materials, reagents and reaction conditions. Therefore, it also has the advantages of low production cost, easily available raw materials, short production cycle and good quality controllability. However, the principle of this method is to combine Fe^3+^ with sodium citrate through oxygen bridge or hydroxyl bridge to form ferric citrate polymer, and citric acid will be complexed on the surface of polymeric iron, which may have a certain impact on the prepared PIC (26). Therefore, the application of this method is less, and its specific mechanism needs further study.

#### Ammonium ferric sulfate method

Adding ammonium ferric sulfate solution slowly into acetic acid solution of polysaccharide for complexation reacting for several hours to obtain a sol solution, and finally precipitating and absorbing the polysaccharide iron compound in a gel state, and centrifugally washing until no ferric ions are detected to obtain the polysaccharide iron complex. Sun (27) prepared chitosan-iron complex by ammonium ferric sulfate method. In the preparation process, the pH value of ammonium ferric sulfate solution was controlled to 1.7–1.8, and then the ammonium ferric sulfate solution was poured into chitosan acetic acid solution for complexation reaction for 3–5 h. Finally, the chitosan-iron complex will precipitate in gel state, and the chitosan iron complex can be obtained after centrifugal washing and drying. In this method, ammonium ferric sulfate is used as chelator, and sulfuric acid and ammonia water are used to control the reaction conditions. Therefore, the reagents used in this method are easily available and low in price, so the preparation cost is relatively low. Compared with co-thermal synthesis of FeCl_3_, the reaction time of this method is 3–5 h, and the preparation time is relatively longer. At present, there are few reports on the preparation of plant polysaccharide iron complex by this method, only a few reports on the preparation of chitosan iron complex. Therefore, this method has great research potential in the field of preparation of plant polysaccharide iron complex.

### Composite membrane simulated biomineralization method

Firstly, chitosan acetic acid solutions with different concentrations were prepared, and then added FeCl_3_·6H_2_O crystal to dissolve with the help of ultrasound, poured the solution on a clean glass plate, and dry it in an oven. Put the prepared membrane into the prepared alkali liquor, and let it stand for several days. Taking out, and finally washing and drying with absolute ethyl alcohol and acetone to obtain chitosan iron compound crystals. The final product, PIC, is in nanocrystalline state. Chen et al. (28) used composite membrane to simulate biomineralization to prepare chitosan iron complex, and a series of characterization and determination of iron content using simulated biomineralization method to prepare PIC. Composite membrane simulated biomineralization method prepares the polysaccharide iron complex by simulating biomineralization, which uses fewer chemical reagents compared with a chemical method and does not need severe reaction conditions such as heating. Therefore, this method has the advantages of mild reaction conditions and smaller PIC particles. However, it often takes several days to simulate the mineralization process, so the reaction time of this method will be longer. At present, this method is mainly applied to the preparation of high purity iron complexes such as chitosan (28), and whether this method can be applied to the preparation of plant polysaccharide iron complexes needs further research.

In summary, the current research on the preparation method of PIC is not comprehensive. PIC is generally synthesized by chemical method, or can be prepared by simulating biomineralization composite membrane under mild conditions, and each has its own characteristics. Considering the experimental operation and preparation efficiency, the chemical synthesis method is the majority of the synthetic methods. Ammonium ferric sulfate method and composite membrane simulated biomineralization method have little application in the preparation of plant polysaccharide iron complex at present and have great development potential. Therefore, the preparation method of PIC needs further research and development to provide material basis for the structural characterization and biological activity research of PIC.

## Analysis of physical and chemical properties of PIC

PIC is a complex formed by the reaction of polysaccharides with iron. Since there are many kinds of polysaccharides, the structure of PIC is different. PICs with different structures often have different physicochemical properties (monosaccharide composition, molecular weight, and stability), as well as pharmacological activities (metabolism, safety, and functional effects) (7). In order to fully study the pharmacological activity of PIC, it is necessary to fully analyze its physical and chemical properties. At present, the research methods for physicochemical properties of PIC are similar to those for polysaccharides, mainly including functional group analysis, chemical composition analysis, monosaccharide composition analysis, molecular weight and its distribution analysis, glycosidic bond connection analysis, microstructure analysis, crystal structure analysis, and thermal stability analysis. This paper summarizes and analyzes the methods which are often used (29).

### Method for determination of iron content in PIC

Although there are many kinds of drugs for the treatment of anemia in the market, the main effective ingredient in clinical application is iron, so it is of great significance for the determination of iron in polysaccharide iron. The detection methods of iron content in PIC include O-phenanthroline spectrophotometry, atomic absorption spectrometry and replacement iodometry. In this paper, the principle and characteristics of the above method are briefly analyzed, and the relevant research results are shown in Table 2.

**Table 2:** Related research results of determination method of iron content in PIC.

**Determination method of iron content**	**Compound name**	**Source of polysaccharides**	**Iron content**	**Ref**.
O-phenanthroline spectrophotometry	ABMP-Fe (III)	*Agaricus blazei*	16.24 ± 0.44%	(17)
	TP-Fe (III)	*Taraxacum*	22.7%	(30)
	MP-Fe (III)	Maca	13.31%	(31)
	MFP-Fe (III)	*Fructus Mori*	2.9–4.9%	(32)
	PPI	*Pyracantha*	30.76%	(33)
Atomic absorption spectrometry	GLP-Fe (III)	*Ganoderma Lucidum*	13.22%	(34)
	LBP-Fe (III)	*Lycium barbarum*	36.71 mg/ml	(35)
	EPP-Fe (III)	*Enteromorpha prolifera*	19.6%	(36)
	LBP-Fe (III)	*Lycium barbarum*	36.71 mg/g	(37)
	SMP-Fe (III)	*Stigma maydis*	269.14 mg/g	(38)
	ZJSP-Fe (III)	*Ziziphus jujube*	21.7%	(39)
	UPP-iron (III)	*Ulva Pertusa*	25.65 ± 3.02%	(22)
	SUE-iron (III)	*Ulva prolifera*	20.3%	(40)
Replacement iodometry	POPFe	*Polygonatum odoratum*	30.13%	(41)
	CP-Fe (III)	Corn	39.86–40.17%	(42)
	FTP-Fe (III)	*Fructus Tribuli*	28.5–29.7%	(43)

#### O-phenanthroline spectrophotometry

**Figure 3:**
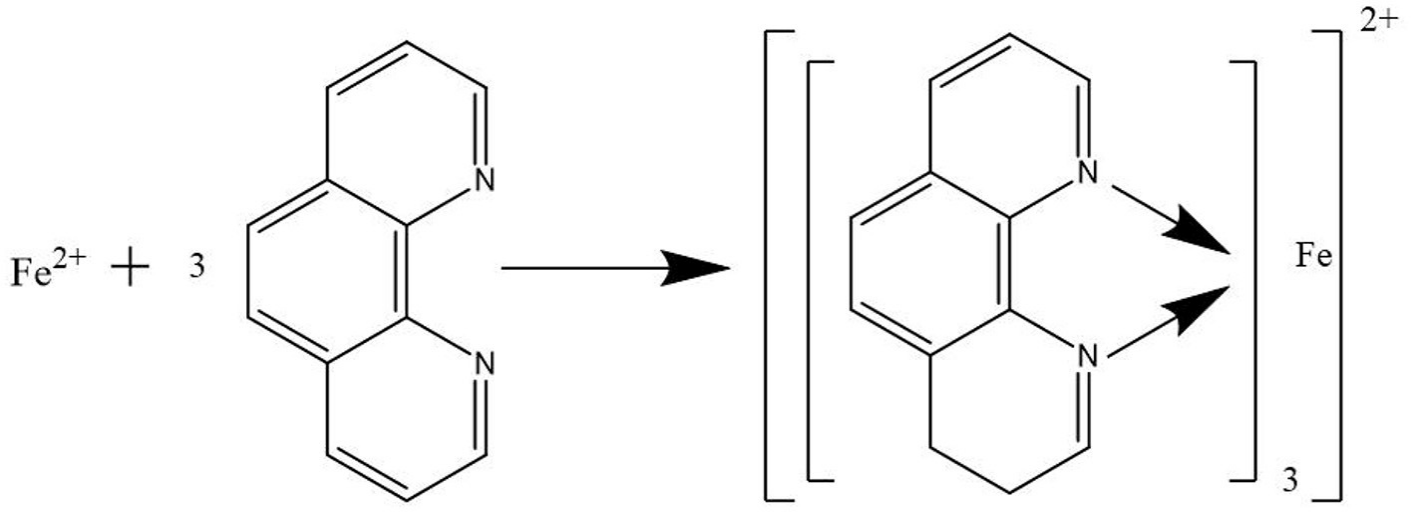
Diagram of iron and phenanthroline reaction.

Iron ion reacted with o-phenanthroline to form a stable orange-red complex in the solution between pH 3–9. The measured wavelength is 510 nm and its molar absorptivity is 1.1 × 10^4^ mol/L, the reaction diagram is shown in Figure 3. If the high valent iron ion is reduced by reducing agent (such as hydroxylamine hydrochloride), the content of high valent iron ion and total iron can be determined by this method, and the content of iron can be determined by o-phenanthroline spectrophotometry. This method has the advantages of small sampling quantity, fast analysis speed, simplicity, accuracy and high sensitivity. However, in the preparation of solution, due to the large number of chemical reagents, these steps can introduce some errors more or less. Therefore, it is necessary to ensure the standardization of the experimental operation (44).

#### Atomic absorption spectrometry

Atomic absorption spectrophotometry (AAS) has become one of the popular analytical methods and has been widely used for the quantitative analysis of related elements in food, medicine, chemical industry and other fields (45). The principle is that after digestion of PIC, the sample solution is injected into the graphite furnace of atomic absorption spectrophotometer. After electro-thermal atomization, iron atoms have specific absorption on the spectral line emitted by the hollow cathode lamp at its maximum absorption wavelength of 248.3 nm. Within a certain range, the absorption value is in direct proportion to the iron content. After compared with the standard curve, the iron content in PIC is determined (34). This method has the characteristics of high sensitivity, simple spectral line, simple and rapid operation, high precision and many kinds of elements, but it also has the disadvantages of expensive equipment and high requirements for experimental conditions (35).


$$\begin{array}{l} \text{Iron binding rate }\left(\text{\%}\right)=\frac{\text{The quality of iron in the sample}}{\text{Sample quality}}\times 100 \end{array}$$

#### Replacement iodometry

Replacement iodometry is an indirect titration method in iodometry, which has the characteristics of convenient experiment, low cost and simple operation, but it also has the disadvantages of low accuracy, low measurement efficiency and easy introduction of human error (46). PIC is a complex of iron ions and polysaccharides, so it cannot be titrated directly. Concentrated hydrochloric acid should be added to dissociate the polysaccharide iron before titration, so that all the iron in the polysaccharide iron was dissociated in the solution in the form of trivalent iron ions (41). Firstly, potassium iodide is added to the test solution (oxidizing substance), and the quantitative iodine is precipitated by oxidation of potassium iodide, and then the iodine is titrated with sodium thiosulfate titration solution, thus the content of the components to be determined is calculated (42). The titration reaction is:


$$\begin{array}{r} \text{Oxidizing substance}+{2\text{I}}^{-}\to {\text{I}}_{2}\\ {\text{I}}_{2}+2{\text{S}}_{2}{\text{O}}_{3}^{2-}\to {\text{S}}_{4}{\text{O}}_{6}^{2-}+2{\text{I}}^{-} \end{array}$$

### Functional group analysis of PIC

Fourier transform infrared spectroscopy (FTIR) is one of the important instruments for functional group analysis, which is simple to operate and selective, and can be used for qualitative analysis of compounds. According to the different structures of samples, through the comprehensive analysis of the position and intensity of the absorption peak, it could determine the corresponding functional groups and structures (22). As we all know, polysaccharide is a kind of polyhydroxy polymer compound, and there are a lot of carboxyl groups on the surface of acidic polysaccharide. These polar groups have strong attraction to Fe^3+^. Many researchers found through infrared spectrum analysis that Fe^3+^ chelated with polysaccharide will not destroy the basic structure of polysaccharide, and PIC also had the basic structure of polysaccharide. Cui et al. (5), Wang et al. (47) and Gao et al. (22), respectively prepared iron complexes of *enteromorpha, astragalus* and *ulva lactuca* by co-thermal synthesis of FeCl_3_ method, and analyzed them in the range of 400–4,000 cm^−1^ by FTIR. The results showed that all of them had the basic characteristic absorption peaks of polysaccharides. This may also be the reason why PIC not only had the function of supplementing iron, but also had the pharmacological activity of original polysaccharide. There were two main changes in the FTIR spectrum of PIC compared with the original polysaccharide. On the one hand, the position and intensity of the peak of original polysaccharide were changed, and on the other hand, the characteristic absorption peak of FeOOH appeared. Zhang et al. (32) prepared three kinds of *mulberry* polysaccharide complexs by co-thermal synthesis of FeCl_3_ method, and analyzed them by FTIR. The results showed that compared with the characteristic absorption bands of the corresponding polysaccharides at 3,450, 1,650 and 1,420 cm^−1^, its intensity decreased obviously, which indicated that Fe^3+^ mainly interacted with the hydroxyl and carboxyl groups of polysaccharides during the synthesis process. In addition, other research results showed that a new characteristic absorption peak appears near 450 cm^−1^, which is mainly due to the formation of Fe-O bond in iron oxide (48, 49). Researchers had a done a lot of research on the configuration of FeOOH formed in PIC. Some researchers found that the iron core of PIC was a kind of β-FeOOH structure. According to the FTIR measurement results of the researchers (50–52) on the polysaccharides iron complex of *Campanumoea javanica*, xylan and *Flammulina velutipe*, the characteristic absorption in the range of 860–900 cm^−1^ and 630–680 cm^−1^ was consistent with the characteristic absorption of β-FeOOH reported in related literature (53–55), indicating that the iron nuclei in these polysaccharide iron may exist in the structure of polymerized β-FeOOH. However, some researchers also found PIC with γ-FeOOH structure. Tang et al. (56) prepared *alginate* polysaccharide iron complex by co-thermal synthesis of FeCl_3_ method, and found that it had characteristic absorption peaks at 1,024 and 750 cm^−1^, which was consistent with the characteristic absorption peaks of γ-FeOOH reported in literature (57), which indicated that the iron core of fucoidan iron complex might be a polymeric γ-FeOOH structure.

To sum up, by comparing the changes of peak shape and peak position by using the infrared spectrum of polysaccharide and PIC, we can judge whether the polysaccharide is chelated with iron ion and whether it is β-FeOOH iron core or γ-FeOOH iron core.

### Thermal stability analysis of PIC

Differential scanning calorimetry (DSC) and thermogravimetric analysis (TGA) are the main instruments for thermal stability analysis of PIC. In the process of programmed heating, the sample will undergo changes such as melting, crystal change and decomposition heat. Then the researchers analyze the data and draw relevant conclusions. The DSC curve is used to study the stability of the complex, and the TG curve represents the weight loss of the sample during the heating process (58).

PIC, as an excellent iron supplement, requires relatively high stability in the process of production, storage and transportation. If the stability is not high and the structure is unstable, the expected iron supplement effect will not be achieved. Its structure is often closely related to its activity, and the change of its structure will also lead to the change of its activity. Therefore, researchers have done a lot of research on the thermal stability of PIC. Liu et al. (59) prepared *auricularia auricula* polysaccharide iron complex by co-thermal synthesis of FeCl_3_ method with FeCl_3_ as chelating agent at 80°C for 2 h, and analyzed it by thermogravimetry and differential thermal analysis. The results showed that *auricularia auricula* polysaccharide iron complex had high thermal stability in the temperature range of 50°C–256°C. This indicated that PIC was still stable at high temperature in chemical synthesis, which was beneficial to the stability of its pharmacological activity. Subsequently, researchers (60–63), respectively prepared *fritillaria* PIC, *spirulina* PIC, *grifola frondosa* PIC and *astragalus* PIC by co-thermal synthesis of FeCl_3_ to study the thermal stability changes of PIC and original polysaccharide. The results of DSC and TG curves showed that PIC has better thermal stability than original polysaccharide. It was possible that Fe^3+^ chelated with the polar groups on the polysaccharide to form a stable β-FeOOH iron core, which made its spatial structure more stable and enhanced its thermal stability. This was conducive to maintaining the stability of the pharmacological activity of PIC during preparation, storage and transportation, making PIC possesed the potential to become an excellent iron supplement.

Differential scanning calorimetry and thermogravimetric analysis can be used to analyze the thermal decomposition and weight loss of polysaccharides and PIC. After the combination of polysaccharides and iron, the trend of weight loss is similar, and the endothermic and exothermic processes are obviously different, which may be due to the fact that polysaccharides are not easy to decompose after binding with iron.

### Microstructure analysis of PIC

Transmission electron microscope (TEM) is one of the main instruments for observing the microstructure of PIC, especially suitable for analyzing the morphology and structure of fine minerals, cryptocrystalline minerals and ultrafine powders. It can collect various signals produced by the interaction between the sample and the high-energy electron beam for high-resolution imaging, and combine with electron diffraction and spectral measurement to characterize the skeleton and guest species, in order to obtain chemical information (64).

Compared with other structural analysis methods of PIC, TEM has more intuitive advantages, and can directly observe the surface micromorphology of PIC, which is helpful for us to fully understand the chelating morphology of Fe^3+^ and polysaccharide in PIC by combining with other structural analysis methods (32). Many researchers have made efforts to do research in this area, and some studies have shown that the microstructure of PIC is a polysaccharide-iron layered nanostructure. Tan and Wang (65) and Shi et al. (66) prepared *ophiopogonis* polysaccharide iron complex and *ulva pertusa* polysaccharide iron complex by co-thermal synthesis of FeCl_3_ method, respectively, and observed their microstructure. The results showed that PIC was distributed in layers, the darker part indicated the strong absorption of electron beam by the iron core, and the lighter part is the edge of its polysaccharide, which also confirmed the layered nanostructure of polysaccharide-iron. At the same time, some researchers put forward the core-shell structure on the basis of PIC layered structure, and studied it. The structure of galactomannan iron complex was analyzed by Huang et al. (67). It could be seen by transmission electron microscope that galactomannan iron complex had a regular sphere with a fuscous nucleus and a tinted shell, which provided more evidence for the hypothesis that the structure of galactomannan iron complex was core-shell structure. At present, the widely recognized microstructure of PIC was a layered core-shell structure, the outermost layer was the shell layer formed by polysaccharides, and the inner part was the iron core aggregated in the form of FeCOOH (62). At the same time, combined with the above mentioned FTIR analysis, it could be speculated that the core-shell structure was that Fe^3+^ was connected with hydroxyl and carboxyl groups on polysaccharide in the form of coordination bonds, thus forming a stable spatial structure.

It was reported that due to the synergistic effect of iron (III) and polysaccharide iron (III), the surface morphology of polysaccharides was different from that of polysaccharide iron. TEM could directly show the surface micro-morphology of the combination of polysaccharide and iron. PIC was a complex formed by polysaccharides wrapped on the surface of iron core with the iron core as the center.

### Crystal structure analysis of PIC

X-ray diffraction (XRD) is an important method for analyzing the crystal structure of PIC. Because of its different chemical composition and structural parameters, any crystal substance will produce different diffraction patterns when X-rays pass through the crystal, that is, they all have specific XRD patterns, which are like human fingerprints, it is the main sign to identify the structure and category of substances (68–70).

Polysaccharides, the most abundant biopolymers on Earth, are one of such systems that do not yield macroscopic single crystals but polycrystalline aggregates of smaller crystallites. This kind of polycrystalline property presents a challenge, which promotes the research on the crystal structure of polysaccharides (71). Many researchers (49, 72) have conducted a large number of studies on the crystal form changes of PIC compared with the original polysaccharide, and they found that there was no sharp absorption peak of PIC, and the diffraction pattern was similar to the overall morphology of the original polysaccharide, indicating that the basic skeleton of the polysaccharide was not damaged, which was consistent with the analysis result in the above mentioned FTIR. Therefore, it was possible that PIC still had the basic activity of the original polysaccharide. In addition, compared with protopolysaccharide, the diffraction pattern of PIC mainly changed in two aspects, on the one hand, the peak shape widened, and on the other hand, the characteristic diffraction peak of iron appeared (23). Zhang et al. (32) and Xu et al. (62), respectively prepared *mulberry* PIC and *grifola frondosa* PIC by co-thermal synthesis of FeCl_3_ and analyzed their crystal morphology. It was found that the diffraction peak of PIC widened, and the characteristic diffraction peaks of iron appeared at the diffraction angles of 15°, 35° and 60°. This showed that the formation of PIC promoted the development of polysaccharide to amorphous direction, and made its particles smaller, which was beneficial to its role of iron supplementation.

The above results showed that the basic skeleton of polysaccharide in PIC was not destroyed, and its XRD diffraction peaks became wider, showing amorphous state, and characteristic diffraction peaks appear at diffraction angles of 15°, 30° and 60°. According to the XRD analysis, not only the binding mode of polysaccharide and iron can be known, but also the crystallinity and structure can be judged according to the position and relative intensity of the peak. The peak shape is round shield, the crystallinity is low, the rigid structure is poor, and the amorphous structure; the peak shape is sharp, has a certain degree of crystallinity, and has a certain spatial structure.

### Other methods

The above mentioned analysis methods of PIC's physical and chemical properties are widely reported, and some researchers use other methods to analyze it. Su et al. (73) determined the structural characteristics of 8 kinds of polysaccharide iron by Mossbauer spectroscopy, the results showed that the iron in the PIC was trivalent iron with high spin and in a weak octahedral state. Zhang et al. (32) analyzed the monosaccharide composition of *mulberry* polysaccharides iron complex by high performance liquid chromatography (HPLC). The results showed that the monosaccharide composition of *mulberry* PIC did not change, but its proportion was obviously different from that of the original polysaccharide. It was speculated that Fe^3+^ chelated with hydroxyl and carboxyl groups in polysaccharide, resulting in the change of its proportion, which might also affect the pharmacological activity of PIC. At the same time, scanning electron microscope was used to better observe its microscopic findings, and it was found that polysaccharide chelated with iron and then became rod-shaped, which was consistent with the results of reference (20).

## Pharmacological activity and clinical application of PIC

At present, most of the pharmacological action of PIC (mainly for the treatment of iron deficiency anemia and hemolytic anemia animal test) is still in the study, the clinical application of some, it is mainly used for iron deficiency anemia in children, adult iron-deficiency anemia is iron deficiency anemia, pregnant women, iron deficiency anemia, renal anemia in the perinatal period of treatment (9). At the same time, PIC can scavenge free radicals and inhibit cell proliferation. In order to promote the product development and clinical application of PIC, we systematically reviewed the pharmacological activities of PIC, and provided reference for the development and utilization of PIC drugs and health products.

### Pharmacological activity of PIC

#### Absorption of PIC

There are no free Fe^2+^ and Fe^3+^ in the PIC. So the PIC is generally absorbed by the body as a molecule. Fe^3+^ is generally dissolved slowly in different acidic and alkaline environments of the human body, and the dissolution time is generally about 6 h. Fe^3+^ is reduced to Fe^2+^ by ascorbic acid, which is absorbed and utilized by the human body (52).

Wang et al. (20) simulated gastrointestinal absorption through *in vitro* digestion test and found that *inonotus obliquus* polysaccharide iron had good bioavailability in simulated gastrointestinal tract and could be used as oral iron supplement. Zhou et al. (74) used isotope tracer method to measure the concentration of ^59^Fe-corn polysaccharide iron in plasma, and the results showed that its pharmacokinetic model in rats was consistent with the two-compartment model, mainly distributed in gastrointestinal tract, and the iron in PIC could enter blood cells after being absorbed by the body for utilization. Zhou and Shen (75) compared the pharmacokinetic parameters of corn polysaccharide iron in rats and beagles, and found that the absorption of corn polysaccharide iron in rats and beagles was significantly different, indicating that the absorption of iron was related to the species and state of experimental animals, providing reference for future clinical application. Cheng et al. (52) prepared the iron complex of *flammulina velina* polysaccharide and studied its absorption *in vitro*. The results showed that the complex had better solubility and stability than ferrous sulfate *in vitro* digestion model. In cell test, the complex showed lower cytotoxicity and better absorption, which could be used as a new ferricarrier for further study.

#### Antioxidant effect of PIC

A series of reactive oxygen species, such as superoxide anion, hydroxyl radical and hydrogen peroxide, are produced during normal metabolism and growth of cells, which can destroy biological macromolecules such as proteins and nucleic acids in human body, resulting in the occurrence of diseases and affecting human health (76–78). Antioxidants slow or prevent the production of excess free radicals and reactive oxygen species and protect the body from damage. As an antioxidant, PIC has stable structure and no side effects (79). Researchers prepared a variety of PIC using co-thermal synthesis of FeCl_3_ method and studied their antioxidant effects. The antioxidant activity indicators and research results were shown in Table 3.

**Table 3:** *In vitro* antioxidant activity indexes and research results of PIC.

**Name**	**Source**	**Results and subjects of *in vitro* free radical scavenging capacity experiments**	**Ref**.
GP-Fe (III)	*Garlic*	DPPH: Vc(K_max_≈90.13%)>PS-Fe(K_max_≈21.15%)>PS(K_max_≈17.16%) ${\text{O}}_{2{•}^{-}}$: Vc(K_max_≈91.26%)>PS-Fe(K_max_≈43.58%)>PS(K_max_≈11.55%) Lipid peroxidation.: Vc(K_max_≈90.15%)>PS-Fe(K_max_≈63.83%)>PS(K_max_≈52.15%) The above K_max_ is at 3.2 mg/mL	(12)
Xyl-Fe (III)	Xylan	DPPH: Vc(K_max_≈90.12%)>Xyl-Fe(K_max_=73.44%)>Xyl(K_max_≈39.85%) •OH: Vc(K_max_≈98.15%)>Xyl-Fe(K_max_=30.95%)>Xyl(K_max_≈20.15%) ${\text{O}}_{2{•}^{-}}$: Vc(K_max_≈98.56%)>Xyl-Fe(K_max_=22.72%)>Xyl(K_max_≈18.85%) The above K_max_ is at 2 mg/mL.	(51)
SP-Fe (III)	Seaweed algae	•OH: SP-Fe(-)>SP(-)	(48)
FUP-Fe (III)	*Fritillaria ussuriensis*	DPPH: Vc(K_max_≈93.12%)>FUP-Fe(K_max_=68.96%)>FUP(K_max_≈42.15%) •OH: Vc(K_max_≈91.15%)>FUP-Fe(K_max_=46.88%)>FUP(K_max_≈38.15%) ${\text{O}}_{2{•}^{-}}$: Vc(K_max_≈98.26%)>FUP-Fe(K_max_=57.28%)>FUP(K_max_≈23.55%) The above K_max_ is at 8 mg/mL	(60)
SP-Fe (III)	*Spirulina*	DPPH: Vc(EC_50_=0.018 g/L)>SP-Fe(EC_50_=1.480 g/L)>SP(EC_50_=2.840 g/L) •OH: Vc(EC_50_=0.021 g/L)>SP-Fe(EC_50_=1.070 g/L)>SP(EC_50_=2.970 g/L) ABTS^+^•: Vc(EC_50_=0.047 g/L)>SP(EC_50_=1.360 g/L)>SP-Fe(EC_50_=1.766 g/L)	(61)
GFP-Fe (III)	*Grifola frondosa*	DPPH: Vc(EC_50_=0.004 mg/mL)>GFP(EC_50_=0.653 mg/mL)>GFP-Fe(EC_50_=3.123 mg/mL) •OH: Vc(EC_50_=0.014 mg/mL)>GFP-Fe(EC_50_=1.134 mg/mL)>GFP(Ec50=3.302 mg/mL) ABTS^+^•: Vc(EC_50_=0.071 mg/mL)>GFP(EC_50_=4.433 mg/mL)>GFP-Fe(EC_50_=19.838 mg/mL)	(62)
APS-Fe (III)	*Astragalus membranaceus*	DPPH: APS(EC_50_=0.4805 mg/mL)>APS-Fe(EC_50_=2.2739 mg/mL) •OH: APS(EC_50_=2.318 mg/mL)>APS-Fe(EC_50_=2.752 mg/mL) ABTS^+^•: GFP(EC_50_=2.542 mg/mL)>GFP-Fe(EC_50_=11.928 mg/mL) ${\text{O}}_{2{•}^{-}}$: APS(EC_50_=1.108 mg/mL)>APS-Fe(EC_50_=4.894 mg/mL)	(63)
GLP-80-Fe (III)	*Glehniae Radix*	DPPH: Vc(IC_50_=0.005 mg/mL)>GFP-Fe(IC_50_=3.79 mg/mL)>GFP(IC_50_=9.06 mg/mL) •OH: Vc(IC_50_=0.072 mg/mL)>GFP-Fe(IC_50_=1.46 mg/mL)>GFP(IC_50_=4.08 mg/mL)	(80)

1,1-diphenyl-2-picrylhydrazyl radical (•DPPH); hydroxyl radical (•OH); superoxide anion radical (${\text{O}}_{2{•}^{-}}$); 2,2'-azino-bis(3-ethylbenzothiazoline-6-sulfonic acid) radical (ABTS^+^•); concentration for 50% of maximal effect (EC_50_); median inhibition concentration (IC_50_).

#### Iron supplement effect of PIC

Iron deficiency anemia has become a common disease bothering modern people, which puts forward requirements for iron supplementation agents with excellent effect and small side effects (81, 82). The inorganic iron-supplementing agents such as ferrous sulfate and ferrous chloride have strong side effects on human bodies although the iron content is high. As an iron supplement, PIC has little side effect on mucosa and intestinal tract, can be absorbed in molecular form, and has the dual role of iron supplement and nutrition (24). Therefore, PIC has great development potential and research value in the field of iron supplements.

Zhang (83) studied the clinical effects of different iron supplementation methods applied in the long-term maintenance of iron supplementation in the treatment of renal anemia with end-stage renal disease. The results showed that intravenous drip of sucrose iron complex or oral PIC could effectively maintain the iron reserve and stabilize the hemoglobin level of patients with renal anemia of end-stage renal disease, and had a good effect of iron supplementation. Wang et al. (84) modified the iron structure of the gonadal polysaccharide of sulfated abalone to prepare the PIC, and studied the iron release rate of the PIC by simulating the digestion process of human gastrointestinal tract. The results showed that the PIC had a good iron supplement efficiency and showed better solubility and stability in the presence of polyphenols/trypsin compared with FeCl_3_, thus it has the potential to be used as a new iron supplement. Li et al. (40) modified the iron structure of low molecular weight sulfate *ulva* polysaccharide to prepare PIC, and established IDA animal model to study the effect of iron supplementation. The results showed that the PIC could effectively restore hemoglobin, red blood cells, serum iron and erythropoietin to normal levels, and had a good effect on iron supplementation. Cui et al. (85) synthesized the iron complex of *enteromorpha* polysaccharide and found that the complex could improve blood indexes and organ indexes of rats with iron deficiency anemia, and had a certain inhibitory effect on iron deficiency anemia, which could be developed as a new iron fortifier. Ganie et al. (86) prepared the iron complex of guar gum, and found that the complex showed the potential to restore the hematological index of albino rats, and had a certain positive effect on improving the growth of iron deficiency anemia rats. Zhang et al. (49) established a mouse model of iron deficiency anemia to study the iron supplementation effect of porphyra PIC. The results showed that the compound could increase red blood cell count, hemoglobin, serum iron, spleen index, spleen weight and body weight of iron-deficiency anemia mice, which is expected to develop into a new iron supplement with synergistic effect on anemia.

#### Antitumor effect of PIC

Cancer, also called malignant tumor, is a disease caused by abnormal cell growth and proliferation mechanism, mainly caused by irregular cell division (87). A large number of studies have reported that natural polysaccharides have shown good antitumor activity in animal experiments *in vivo*, with less toxic and side effects (88). The inherent structure of polysaccharide may partially limit its antitumor activity. Structural changes caused by molecular structure modification may improve and change the pharmacological activity of polysaccharides and promote the application of polysaccharides (89). Therefore, it is of great significance to study the antitumor activity and mechanism of PIC.

The antitumor mechanism of PIC is very complex. Some studies have shown that PIC can directly act on tumor cells and induce apoptosis, thus inhibiting the proliferation of tumor cells. Li et al. (33) studied the optimum synthesis process of *Pyracantha* polysaccharide iron (PPI) complex by response surface methodology (RSM) and its antitumor activity. The results showed that PPI had better cytotoxicity on human oval cells (SKOV3), which could induce apoptosis of human oval cells and change gene expression. PIC can also induce apoptosis by regulating mitochondrial apoptosis pathway. Feng and Zhang (13) reported that *C. pilosula* polysaccharide iron (CPPI) could inhibit the proliferation of A2780 cells, depolarize mitochondrial membrane potential (MMP), and induce apoptosis and DNA damage of ovarian cancer cells in a dose-dependent manner. Reactive oxygen species (ROS) is a highly active chemical substance containing oxygen free radicals. The dynamic balance of reactive oxygen species (ROS) production and scavenging is the key to the dynamic balance of redox. The excessive production of ROS can promote the apoptosis of tumor cells. Because of gene changes, cancer cells will proliferate inappropriately and get out of control (90). Xian et al. (48) compared the effects of Sulfated polysaccharide (SP) and sulfated polysaccharide iron complex (SPIC) on ROS production in THP-1 cells. It was found that SP reduced the production of ROS, but its iron chelating agent SPIC could effectively induce the production of ROS, showing the application potential of SPIC in antitumor activity. At the same time, PIC can indirectly induce lymphocyte proliferation, enhance immune regulation ability, and thus realize tumor inhibition. Zhang et al. (61) evaluated the immunomodulatory activity of *Spirulina* polysaccharide (SP) and *Spirulina* polysaccharide-Fe (SP-Fe) by lymphocyte proliferation method. The results showed that SP-Fe could promote the proliferation of lymphocytes, and had good immunomodulatory activity and certain antitumor potential.

The above studies show that PIC has certain antitumor activity, but its specific mechanism of action is relatively complex. On the one hand, PIC can directly act on tumor cells, induce apoptosis and DNA damage, and also regulate mitochondrial apoptosis pathway, promote the release of ROS and inhibit the proliferation of tumor cells. On the other hand, it can also promote lymphocyte proliferation, enhance the body's immunity and kill tumor cells through immune regulation. Therefore, PIC has less toxic and side effects and good antitumor effect. It is expected to become a new antitumor drug. However, the specific mechanism of PIC killing tumor cells is still unclear and needs further study.

### Clinical application of PIC

PIC not only has a variety of pharmacological activities of polysaccharide itself, but also has the effect of enriching blood. It has been widely used in clinical treatment due to its good absorption effect, good patient compliance and no toxic or side effects. It is mainly used for treating anemia symptoms caused by pregnancy, nephropathy and diabetes (91). The current clinical cases of PIC in the treatment of diseases were analyzed in order to provide a theoretical basis and research direction for the product development of PIC.

Li et al. (92) used PIC capsules combined with multivitamin tablets to treat iron deficiency and iron deficiency anemia in pregnant women and women of childbearing age, and achieved good clinical effects according to monitoring the recovery and increase of hemoglobin and serum protein. Yuan (93) conducted a clinical study and found that the treatment of pregnancy anemia, with fewer adverse reactions, can effectively improve the levels of Hb, SF in pregnant women, natural delivery rate and clinical treatment effect, and reduce the incidence of neonatal distress, which is worthy of clinical promotion and application. Xia et al. (94) explored the effect of the application of PIC in the treatment of iron deficiency anemia on serum ferritin level of patients. Using ferrous sulfate particles as the control group, the study results showed that PIC had a more significant effect on IDA treatment, which could promote the synthesis of serum ferritin and inhibit the release of serum transferrin receptors. Li et al. (95) found that the treatment of PIC combined with hemodialysis and recombinant human erythropoietin in patients with chronic renal failure complicated with renal anemia has significant curative effect, and can greatly improve the patient's clinical indicators, optimize the patient's renal function, reduce the occurrence of adverse reactions, and improve the patient's serum calcium and phosphorus indicators. Gong et al. (96) found in the clinical study that with ferrous succinate tablets as the control group, the treatment effect of polysaccharide iron capsules was more obvious for patients with iron deficiency anemia during pregnancy, which could effectively improve the blood biochemical indicators of patients and promote the improvement of symptoms, with high safety. Zou (97) explored the effect of using PIC capsule combined with Shengxuening tablet in the treatment of renal anemia of peritoneal dialysis. The study found that PIC capsule combined with Shengxuening tablet had satisfactory effect and high safety in the treatment of renal anemia of peritoneal dialysis. Chen and Dai (98) found in the clinical study that shengxuebao mixture combined with PIC can effectively improve the anemia of patients with postpartum anemia with fewer adverse reactions. Chen et al. (99) found in the clinical study that oral PIC combined with erythropoietin could significantly increase the hemoglobin compliance rate of patients with maintenance hemodialysis compared with erythropoietin alone, which is worthy of clinical promotion and application.

## Study on structure-activity relationship of PIC

The pharmacological activity of PIC is highly related to its chemical composition, structural characteristics and spatial conformation (52). However, due to the high molecular weight, complex structure and easy change of spatial conformation of PIC, there are few related studies at present and the structure-activity relationship can't be clearly explained, and even different researchers may draw different conclusions. (1) Relationship between iron and pharmacological activity of PIC. Generally, iron is an important active source of PIC as a blood tonic, but does the chelation of iron and the increase of its content also mean that the pharmacological activity of original polysaccharide is improved? Some researchers have found that PIC had better antioxidant activity than original polysaccharide (100). This indicated that the increase of PIC activity should be related to iron chelation and the increase of content. This may be due to the change of the space structure of polysaccharide after it was combined with iron, and the coordination part cooperates with the exposed active groups to promote the interaction with free radicals, thus enhancing the ability of scavenging free radicals (80). However, Wang et al. (91) prepared *Rosa roxburghii* polysaccharide iron complex and found that its antioxidant activity was lower than that of the original polysaccharide. This may be because it forms a stable β-FeOOH iron core structure, which weakened the hydrogen supply capacity and thus weakened the scavenging activity of DPPH free radicals. This indicated that the combination of iron and the increase of iron content did not necessarily lead to the increase of antioxidant capacity, but the explanation of this finding was still unclear. Therefore, researchers should not only pay attention to the increase of iron content in PIC. (2) Relationship between physicochemical properties and pharmacological activity of PIC. Monosaccharide composition is an important factor in the structure-activity relationship. The content of glucuronic acid (GlcA) in acidic polysaccharides was positively correlated with its antioxidant activity, and the higher the content of GlcA, the better the antioxidant activity. In neutral polysaccharides, the higher the content of glucose (Glc), the better the antioxidant activity (101). Peng et al. (102) found that the original monosaccharide composition will not be changed after the *flammulina velutipes* scraps polysaccharide (FVSP) chelates with iron, but the proportion of monosaccharide composition will be changed. Because of the different configuration of C-2 hydroxyl, the binding constant of mannose with metal ions was higher than that of other monosaccharides such as glucose and galactose, which made mannose bound by iron, thus increasing the content of glucose. It was speculated that this may also be the reason for the higher antioxidant activity of FVSP-iron (III), indicating that the monosaccharide composition affects PIC activity. At the same time, Yao et al. (103) found that the stability of PIC was better than that of original polysaccharide, which may be because polysaccharide and iron form a stable β-FeOOH iron core, making its spatial structure more stable, which was conducive to the transportation of PIC *in vivo* and reaching the absorption site. However, there are few studies on the structure-activity relationship of PIC, such as antitumor and other activities. It is necessary to further study the structure-activity relationship of PIC in the future to provide theoretical basis for its practical application.

## Product development of PIC

PIC, with its low toxicity and significant pharmacological activity, plays an important role in medical and biological health care. More and more PICs were developed for modern food supplements and clinical drugs (104). Due to its above-mentioned pharmacological activities, PIC can be applied to the fields of health products, functional foods and drugs. In the international market, there are currently about six drugs developed and marketed with PIC as the active ingredient, which are mainly used as iron supplements and for the treatment of iron deficiency anemia (8). However, no relevant reports on the functional foods of PIC were retrieved. Therefore, the applications of PIC in the field of health care products is relatively rare, and it still has a great development potential, which needs to be further expanded in the field of health care products and drugs.

**Figure 4:**
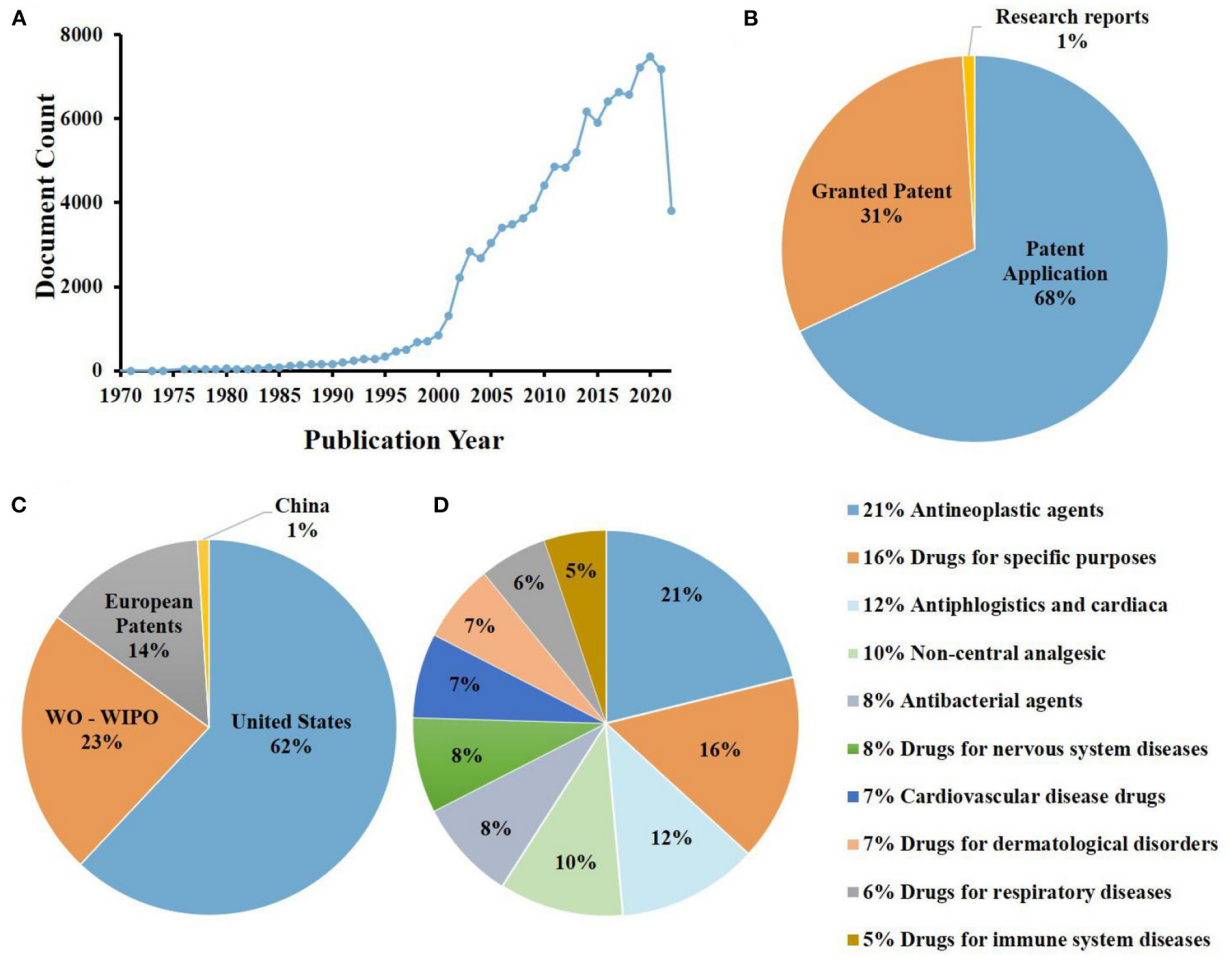
Analysis of 108,797 patents browsed in www.lens.org using the search terms “Polysaccharide iron complex”: **(A)** patent applications per years; **(B)** document type; **(C)** jurisdictions (WO-WIPO, world intellectual property organization); **(D)** central product classification.

The analysis of the PIC patent situation helps us to have a more full understanding of the current development, and provides a theoretical basis for the future development of PIC applications. A detailed analysis of PIC patent status is shown in Figure 4. At present, there are about 108,797 patents related to PIC in the world, among which the proportion of patent applications is the largest, accounting for 68%, the proportion of granted patent is smaller, at 31%, and the proportion of research reports is the smallest, only 1%. Among them, the United States has the most patents, accounting for about 62%, the world intellectual property organization (WO-WIPO) of several patents, accounting for about 23%, and China and other countries the smallest proportion, accounting for about 1%. PIC related patents mainly focus on drug research and development, among which antineoplastic agents accounted for a maximum of 21%. Although related drugs have been applied in clinical research, there still exist such problems as small number of application products, single application field, and limited active application. In the future research, we need to strengthen the research in this area, and give full play to the application potential of PIC.

## Conclusions and perspectives

As mentioned above, we described the preparation, structural characteristics and pharmacological activity of plant polysaccharide-iron complex systematically and in detail. Chemical synthesis is the most widely used method in the preparation of PIC. This method has the advantages of low cost, easily available raw materials, short production cycle, etc., but it also has the disadvantages of heavy environmental pollution and poor recycling. Therefore, the preparation method of PIC still needs further study in the future. PIC is the chelation of polysaccharide and iron ions, and it is β-FeOOH iron core or γ-FeOOH iron core. The structure of polysaccharide is often closely related to its activity. The amount of iron in PIC and the chelation mode between iron and polysaccharide groups all affect its antioxidant activity. Excessive iron content may inhibit the antioxidant activity of polysaccharide. Therefore, researchers should not only pay attention to improving the iron content of PIC. In addition, the combination of Fe^3+^ and polysaccharide will also affect the proportion of monosaccharide composition, thus changing the pharmacological activity. PIC has high water solubility and is absorbed in molecular form, and its absorption rate is equivalent to that of ferrous sulfate, which makes it a new type of iron supplement with great development potential.

Although the development of PIC and related scientific research have made great progress, there are still some problems, and some suggestions are put forward for these problems. (1) The preparation method of plant polysaccharide iron complex is relatively simple, and about 90% of the studies are based on the co-thermal synthesis of FeCl_3_, almost no ammonia ferric sulfate method and composite membrane simulated biomineralization method are used. At present, only small molecular chitosan-iron complex has been prepared by these two methods. Therefore, researchers should pay attention to the research of these two methods to make up for the deficiency of the existing methods. (2) More than 80% of the related researches focus on antioxidation, iron supplementation and antitumor. In contrast, there are a few studies on other pharmacological activities of PIC. Therefore, researchers should pay more attention to the study of hypoglycemic, heavy metal scavenging and other effects. (3) The biological activity of polysaccharides is highly dependent on its special structure. Although a few reports show that the biological activity of PIC is closely related to its special structure, its active conformation and the relationship between structure and pharmacological activity have not been fully established. Therefore, it is necessary to strengthen the research on the structure-activity relationship of PIC, which helps researchers design PIC with stronger activity and lower toxicity through structural modification. (4) At present, about six PIC drugs have been put on the market for use in the world, while the plant polysaccharide iron complex is still in the research stage. And there are few products on the market, no health products or functional foods, so the research on practical application is insufficient. Therefore, we offer two conjectures about the current research and development direction of iron supplement. One is to find ligands with higher safety and more controllable iron release, such as plant polysaccharides with pharmacological activity, and the other is to improve the stability of products and the similarity between products and ferritin.

## Author contributions

YJ and LW designed and conceived the study. YJ, SZ, and ML performed the main content. SZ, YZ, and ML analyzed the data and drafted the manuscript. RZ, HZ, and SZ contributed to the writing of the manuscript. YJ and DZ provided the funding and resources. All authors revised and approved the submitted version of the manuscript.

## Funding

This work was supported by the 333 Talent Project Program of Hebei (No. C20221090); the S&T Program of Hebei (No. H2020208032).

## Conflict of interest

The authors declare that the research was conducted in the absence of any commercial or financial relationships that could be construed as a potential conflict of interest.

## Publisher's note

All claims expressed in this article are solely those of the authors and do not necessarily represent those of their affiliated organizations, or those of the publisher, the editors and the reviewers. Any product that may be evaluated in this article, or claim that may be made by its manufacturer, is not guaranteed or endorsed by the publisher.
